# Recessive *NLRC4*-Autoinflammatory Disease Reveals an Ulcerative Colitis Locus

**DOI:** 10.1007/s10875-021-01175-4

**Published:** 2021-11-16

**Authors:** Annemarie Steiner, Thomas Reygaerts, Alessandra Pontillo, Isabella Ceccherini, Jonas Moecking, Fiona Moghaddas, Sophia Davidson, Francesco Caroli, Alice Grossi, Fabio Fernandes Morato Castro, Jorge Kalil, Florian N. Gohr, Florian I. Schmidt, Eva Bartok, Thomas Zillinger, Gunther Hartmann, Matthias Geyer, Marco Gattorno, Leonardo Oliveira Mendonça, Seth L. Masters

**Affiliations:** 1 Inflammation Division, The Walter and Eliza Hall Institute of Medical Research, 1G Royal Parade, Parkville, VIC 3052 Australia; 2 Department of Medical Biology, The University of Melbourne, Parkville, VIC 3010 Australia; 3 Institute of Structural Biology, Medical Faculty, University of Bonn, 53127 Bonn, Germany; 4 Immunogenetic Laboratory, Department of Immunology, Biomedical Science Institute, Universidade of São Paulo, São Paulo, Brazil; 5 Laboratory of Genetics and Genomics of Rare Diseases, IRCCS Istituto Giannina Gaslini, 16147 Genoa, Italy; 6 Department of Clinical Immunology and Allergy, The Royal Melbourne Hospital, Parkville, VIC 3052 Australia; 7 Division of Clinical Immunology and Allergy, Department of Internal Medicine, Universidade of São Paulo, São Paulo, Brazil; 8 Institute of Innate Immunity, Medical Faculty, University of Bonn, 53127 Bonn, Germany; 9 Department of Microbiology and Immunology, The University of Melbourne, Parkville, VIC 3010 Australia; 10 Institute of Clinical Chemistry and Clinical Pharmacology, University Hospital Bonn, 53127 Bonn, Germany; 11 Unit of Experimental Immunology, Department of Biomedical Sciences, Institute of Tropical Medicine, Antwerp, Belgium; 12 Institute of Immunology, Philipps-University Marburg, BMFZ, 35043 Marburg, Germany; 13 German Center for Infection Research (DZIF), Partner Site Bonn-Cologne, Cologne, Germany; 14 Center for Autoinflammatory Diseases and Primary Immunodeficiencies, IRCCS Istituto Giannina Gaslini, 16147 Genoa, Italy; 15 Center for Rare and Immunological Disorders, DASA-Hospital 9 de Julho, São Paulo, Brazil

**Keywords:** NLRC4-associated autoinflammatory disease, inflammasome, ulcerative colitis

## Abstract

**Purpose:**

NLRC4-associated autoinflammatory disease (NLRC4-AID) is an autosomal dominant condition presenting with a range of clinical manifestations which can include macrophage activation syndrome (MAS) and severe enterocolitis. We now report the first homozygous mutation in *NLRC4* (c.478G > A, p.A160T) causing autoinflammatory disease with immune dysregulation and find that heterozygous carriers in the general population are at increased risk of developing ulcerative colitis.

**Methods:**

Circulating immune cells and inflammatory markers were profiled and historical clinical data interrogated. DNA was extracted and sequenced using standard procedures. Inflammasome activation assays for ASC speck formation, pyroptosis, and IL-1β/IL-18 secretion confirmed pathogenicity of the mutation in vitro. Genome-wide association of NLRC4 (A160T) with ulcerative colitis was examined using data from the IBD exomes portal.

**Results:**

A 60-year-old Brazilian female patient was evaluated for recurrent episodes of systemic inflammation from six months of age. Episodes were characterized by recurrent low-grade fever, chills, oral ulceration, uveitis, arthralgia, and abdominal pain, followed by diarrhea with mucus and variable skin rash. High doses of corticosteroids were somewhat effective in controlling disease and anti-IL-1β therapy partially controlled symptoms. While on treatment, serum IL-1β and IL-18 levels remained elevated. Genetic investigations identified a homozygous mutation in *NLRC4* (A160T), inherited in a recessive fashion. Increased ASC speck formation and IL-1β/IL-18 secretion confirmed pathogenicity when NLRC4 (A160T) was analyzed in human cell lines. This allele is significantly enriched in patients with ulcerative colitis: OR 2.546 (95% 1.778–3.644), *P* = 0.01305.

**Conclusion:**

NLRC4 (A160T) can either cause recessively inherited autoinflammation and immune dysregulation, or function as a heterozygous risk factor for the development of ulcerative colitis.

**Supplementary Information:**

The online version contains supplementary material available at 10.1007/s10875-021-01175-4.

## Introduction

NLRC4-associated autoinflammatory disease (NLRC4-AID) is a recently described autosomal dominant condition presenting with a range of clinical manifestations that can include macrophage activation syndrome (MAS) and severe enterocolitis [1, 2]. Here, we report the phenotype of a patient with a homozygous mutation in NLRC4 (A160T).

## Methods

### Genetic Investigations

Informed consent from study subjects was obtained for genetic investigations. Genomic DNA of the patient, healthy parents and the brother was sequenced at the Laboratory of Genetics and Genomics of Rare Diseases “Giannina Gaslini” Institute (Genoa, Italy) for the analysis of selected genes [3]. Next generation sequencing (NGS) was performed, using an Ampliseq design coupled with Ion PGM™ parallel sequencing (Thermo Fisher Scientific, Waltham, MA) and demonstrated the presence of a homozygous variant in the *NLRC4* gene that had only been reported as heterozygous genotype in the healthy population (GnomAD). The variant was confirmed and segregated by Sanger sequencing. Within the analyzed panel of target genes [3], only one other heterozygous variant was identified in the *IL1RN* gene, resulting in a synonymous mutation (c.345C > T, p.D115 =).

### Healthy Donors

Peripheral blood was collected for monocytes isolation from 21 unrelated healthy donors (HCs, 11 males/10 females; 35.4 ± 13.4 years) (Fig. 2a) and 3 HCs (1 male/2 females; 24.7 ± 23.3 years) (Fig. 2b, Suppl. Figure 1a), proceeding from the metropolitan area of São Paulo (SP, Brazil).

### Isolation of Peripheral Blood Monocytes and Monocyte-Derived Macrophages

Peripheral blood mononuclear cells (PBMC) were isolated from 20 ml blood using Ficoll-Paque density gradient centrifugation (GE Healthcare, Chicago, IL), and monocytes were separated from other PBMCs by adhesion in 96-well culture plates (0.8 × 10^6^ PBMC/well) (Corning-Costar, New York, NY). After 2 h, non-adherent cells (mainly lymphocytes) were removed by washing 3 times with phosphate buffer saline (PBS; Sigma-Aldrich, St. Louis, MO), while adherent cells (mainly monocytes) were cultured in RPMI-1640 (Gibco, Thermo Fisher Scientific, Waltham, MA) supplemented with 10% of fetal bovine serum (FBS; Gibco, Thermo Fisher Scientific, Waltham, MA) at 37 °C in 5% CO_2_. To obtain monocyte-derived macrophages (MDMs), monocytes were cultured in RPMI-1640/10% FBS with 25 ng/ml monocyte colony stimulating factor (M-CSF; PeproTech, Rocky Hill, NJ) at 37 °C in 5% CO_2_ for 5 days.

### Inflammasome Activation Analysis in Monocyte-Derived Macrophages

MDMs were challenged with 1 µg/ml bacterial lipopolysaccharide (LPS, *E. coli* strain O111:B4; Sigma-Aldrich, St. Louis, MO) for 4 h with subsequent addition of 1 mM ATP (Sigma-Aldrich, St. Louis, MO) for 15 min or cytoplasmic delivery of 5 µg/ml flagellin from *S. typhimurium* (FLA, InvivoGen, San Diego, CA) in DOTAP liposomes (6 µg DOTAP (Roche)/1 µg FLA) for 2 h before supernatants were collected. Long-term stimulation was performed with 1 µg/ml LPS or 5 µg/ml FLA for 24 h.

### Cytokine Measurements

IL-1β (Biolegend, San Diego, CA and BD Bioscience, San Diego, CA), IL-18 (R&D Systems, Minneapolis, MN), TNF (R&D Systems, Minneapolis, MN), and IL-8 (BD Bioscience, San Diego, CA) were measured in cell culture supernatants by ELISA according to the manufacturer’s protocol.

### Quik Change Mutagenesis of Plasmids

The QuikChange Lightning Site-Directed Mutagenesis Kit (Agilent Technologies, Santa Clara, CA) was used to introduce point mutations in *NLRC4* (c.478G > A, p.A160T and c.512C > T, p.S171F) following the manufacturer’s instructions. As templates, mammalian expression construct pEBOS-mCit-hNLRC4 and pcDNA5/FRT/TO NLRC4-SH plasmids were used. Primer sequences are shown in Suppl. Table 1.

### Cell Culture

HEK293T and HEK293T-ASC-RFP cells [4] were maintained in complete Dulbecco’s modified Eagle medium (DMEM) supplemented with 10% FBS (Sigma-Aldrich, St. Louis, MO) and 100 U/ml Penicillin/100 μg/ml Streptomycin in a humidified incubator at 37 °C and 5% CO_2_. Flp-In 293 T-REx cells (Thermo Fisher Scientific Cat# R78007) were cultured with addition of 50 g/ml zeocin (InvivoGen, San Diego, CA) and 4 μg/ml blasticidin (InvivoGen, San Diego, CA). THP-1 monocytes were cultured in complete RPMI-1640 supplemented with 10% FBS and 100 U/ml Penicillin/100 μg/ml Streptomycin in a humidified atmosphere at 37 °C and 5% CO_2_.

### Generation of Flp-In 293 T-REx Cell Lines Stably Expressing ASC-EGFP and hNLRC4

Stable ASC-EGFP-expressing monoclonal Flp-In 293 T-Rex cells were generated via lentiviral transduction. Second generation lentiviral plasmids psPax2 (0.65 μg) and pMD2.G (0.35 μg) were co-transfected with pRRL pUbC ASC-EGFP (1.5 μg) in HEK293T cells in a 6-well plate format using PEI Max (1 mg/ml, Polysciences, Warrington, PA) at a DNA (mg):PEI (ml) ratio of 1:1. The cells were incubated for 6 h before the medium was replaced and virus-containing supernatant was harvested 48 h post transfection. The Flp-In 293 T-REx cells were inoculated with 250 μl viral supernatant in presence of 10 μg/ml polybrene (Sigma-Aldrich, St. Louis, MO) using a 24-well plate format. After 10 h incubation, the culture medium was replaced. The cells were incubated overnight and then transferred into a 10 cm dish. After overnight incubation, puromycin selection was initiated (1 μg/ml, InvivoGen, San Diego, CA). Medium exchange was performed every 3 days. The cells were cultured until visible single cell colonies appeared. Multiple single colonies were picked and cultured to confluence. Following screening of activation-induced versus baseline ASC specking by flow cytometry, the clone with the highest ratio was selected to subsequently generate isogenic human NLRC4-expressing Flp-In 293 T-REx-ASC-EGFP cells lines using the Flp-In system following the manufacturer’s instructions (Thermo Fisher Scientific, Waltham, MA). Expression of human NLRC4 WT, A160T or S171F with C-terminal Strep2-HA (SH) tag was induced by doxycycline treatment (1 μg/ml, Sigma-Aldrich, St. Louis, MO) for indicated periods. The cells were maintained in complete medium as described above with addition of 4 μg/ml blasticidin (InvivoGen, San Diego, CA), 50 μg/ml hygromycin B (InvivoGen, San Diego, CA), and 1 μg/ml puromycin (InvivoGen, San Diego, CA).

### Time of Flight Inflammasome Evaluation in HEK293T ASC-RFP Cells and Flp-In 293 T-REx ASC-EGFP NLRC4 Cell Lines

To assess inflammasome activation, flow cytometric analysis of time of flight inflammasome evaluation (TOFIE) was performed [5]. The method quantifies the activation-dependent redistribution of apoptosis-associated speck-like protein containing a CARD (ASC) into specks that reflect inflammasome formation. Figure 2c shows HEK293T ASC-RFP cells transiently transfected with 50 ng of mCitrine-tagged pEBOS-hNLRC4 WT, A160T, or S171F using a 24-well plate format. ASC speck formation was quantified 15 h after transfection by flow cytometry. Analysis was performed with FlowJo 10.5 software gating on the total population of mCitrine-expressing cells. Gates were individually adjusted for the not mCitrine-expressing empty vector (EV) control. To assess inflammasome activation in Flp-In 293 T-REx ASC-EGFP cells (Fig. 2e, Suppl. Figure 1d), NLRC4 expression was induced by doxycycline treatment (1 μg/ml) for indicated times. Quantification of ASC speck formation was performed at baseline (Suppl. Figure 1d) as well as after transient transfection with 250 ng of pcDNA-3xFLAG-hNAIP and pcDNA-myc-PrgI expression constructs (Fig. 2e). ASC speck formation of the total population of EGFP-expressing cells was analyzed. Western blot analysis was performed to control for comparable target protein expression.

### Western Blot

Cells were lysed in 1% NP40 buffer (1% NP40, 10% glycerol, 20 mM Tris–HCl (pH7.5), 150 mM NaCl, 1 mM EGTA) complemented with complete protease inhibitors (#11697498001, Roche Biochemicals, Basel, Switzerland) and 1 mM PMSF (Roth, Karlsruhe, Germany) and incubated on ice for 30 min. The lysates were clarified by centrifugation for 15 min at top speed and eluted in SDS-PAGE sample buffer. SDS-PAGE was performed using Novex 4–12% gels (Life Technologies, Carlsbad, CA) with MES running buffer (Thermo Fisher Scientific, Waltham, MA) and subsequently transferred onto polyvinylidene difluoride (PVDF) membranes (Merck Millipore, Kenilworth, NJ). The membranes were blocked in 5% skim milk plus Tris-buffered-saline-Tween 20 (TBST) at room temperature for 1 h and then incubated in primary antibody overnight at 4 °C. Primary antibodies used are α-NLRC4 (rabbit anti-human NLRC4, D5Y8E, Cell Signalling Technologies, Danvers, MA), β-Actin-HRP (C4) (sc-47778, Santa Cruz Biotechnology, Dallas, TX), and monoclonal anti-FLAG M2-peroxidase (HRP) antibody (Sigma-Aldrich, St. Louis, MO).

### Genome Editing by CRISPR/Cas9-Mediated Homology-Directed Repair in THP-1 Cells and Sequencing

THP-1 WT cells were transiently electroporated (Neon Transfection System, Thermo Fisher Scientific,Waltham, MA; 1250 V, 50 ms, 1 pulse, 5 × 10^5^ THP-1 WT cells, 100 µl tips) with 10 μg of the plasmid EF1alpha-Cas9-2A-EGFP/U6-guide RNA targeting NLRC4 and 2.5 μM oligo repair template encoding the nucleotide exchange c.468C > T (silent mutation on protein level) to introduce a *Bsa*I recognition site as well as the patient mutation of interest, c.478G > A which encodes p.A160T (Suppl. Figure 2a, sequences listed in Suppl. Table 1). FACS sorting for EGFP-positive cells was performed 18 h after electroporation and monoclonal cell lines generated by limiting dilution. Total DNA from single cell clones was isolated by Proteinase K lysis (200 μg/ml) in PBND buffer (50 mM KCl, 10 mM Tris–HCl (pH8.3), 2.5 mM MgCl_2_, 0.1 mg/ml gelatin, 0.45% (v/v) NP40, 0.45% (v/v) Tween 20). A subsequent PCR reaction was performed to amplify the genomic *NLRC4* locus and *Bsa*I (NEB, Ipswich, MA) enzymatic digest was performed to screen clones for successful oligo repair template integration. PCR products of selected clones were enzymatically purified with rSAP and Exo1 (NEB, Ipswich, MA) digest and submitted for Sanger sequencing to confirm genotypes on genomic level. Analysis of > 1000 single cell clones identified 1 clone with in-frame insertion of the correct mutation on one allele and 1 bp deletion on the second allele generating a A160T/KO genotype. A matching WT/KO control clone and 3 WT/WT clones carrying one or two functional *NLRC4* WT alleles, respectively, were selected as controls for inflammasome stimulation experiments. Furthermore, a KO/KO clone with homozygous 143 bp deletion inducing a frameshift and premature stop codon was included. Sequences of active *NLRC4* mRNA transcripts were confirmed by Sanger sequencing after RNA isolation and cDNA transcription (method described below), which then served as template for *NLRC4*-specific PCR with exon-spanning primers (Suppl. Figure 2b, sequences listed in Suppl. Table 1).

### Analysis of Relative NLRC4 mRNA Transcript Levels by qRT-PCR

Lysis of THP-1 cell clones was performed with RLT lysis buffer (Qiagen, Germany) and RNA isolated using EconoSpin columns (Epoche Life Science, Missouri City, TX) with on-column DNA digest (DNaseI, Thermo Fisher Scientic, Waltham, MA) following the manufacturer’s instructions. cDNA was synthesized using ReverseAid Transcriptase (Thermo Fisher Scientific, Waltham, MA) according to the manufacturer’s recommendations and subsequently analyzed by qRT-PCR using my-Budget 5 × Eva Green QPCR-Mix II (Bio-Budget Technologies GmbH, Germany). Primer sequences for *NLRC4* and *ACTB* are listed in Suppl. Table 1. Data are presented as ΔΔCt normalized to the *ACTB* and THP-1 WT pool sample.

### THP-1 Cell Stimulation

NLRC4-specific stimulation was performed by priming with TLR1/2 agonist Pam3CSK4 (P3C) at 100 ng/µL (InvivoGen, San Diego, CA) and simultaneous retroviral transduction with pMXsIG_PrgI_GFP in presence of polybrene (8 μg/ml) as previously described [6]. After 24 h, supernatants were collected for quantification of IL-1β, IL-18, and IL-8 cytokine levels by ELISA and cell death was analyzed by flow cytometry following propidium iodide (PI) staining (1 µg/ml, Sigma-Aldrich, St. Louis, MO) of cells. The NLRP3 inflammasome was stimulated with 10 μM Nigericin (InvivoGen, San Diego, CA) for 1 h after 3 h priming with P3C (100 ng/µL).

### Statistical Analysis

Statistical significance was calculated using GraphPad Prism Version 9.0.2. Performed tests are stated in the respective figure legends. Considered *P* values are as follows: *P < 0.05, **P < 0.01, ***P < 0.001, ****P < 0.0001.

## Results and Discussion

**Fig. 1:**
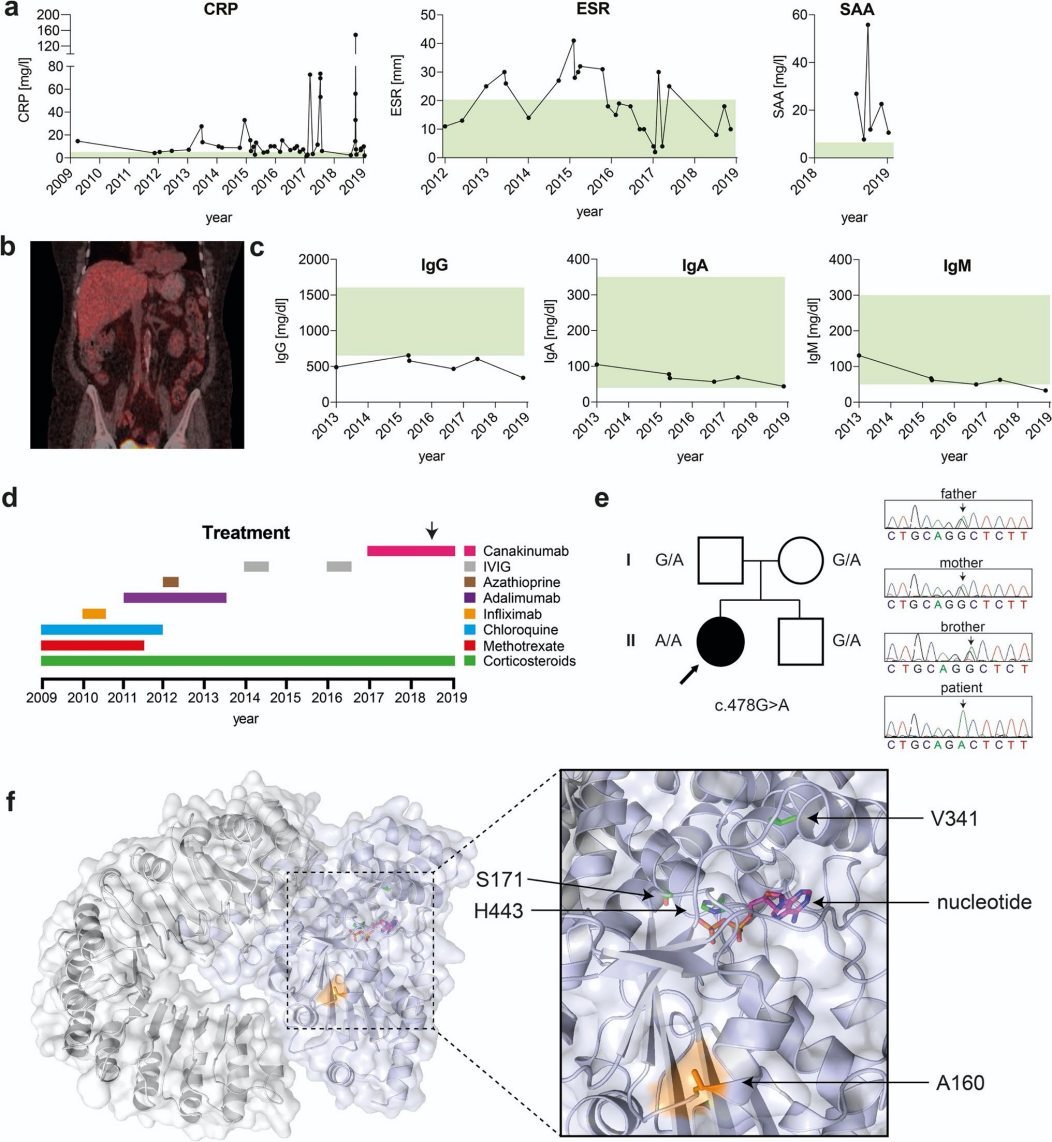
Biochemical analysis of patient samples, treatment timeline, patient pedigree, and structural analysis. (**a**) Increased C-reactive protein levels (CRP, normal range (green): < 5 mg/l), erythrocyte sedimentation rate (ESR, normal range (green): < 20 mm) and serum amyloid A levels (SAA, normal range (green): < 6.4 mg/l) measured in the patient over several years. (**b**) Whole-body PET-CT scan performed during inflammatory episode in 2016 indicates heterogeneous and diffuse bowel glycolytic activity. (**c**) The patient persistently presented with low serum IgG (normal range (green): 650–1600 mg/dl), IgA (normal range (green): 40–350 mg/dl), and IgM (normal range (green): 50–300 mg/dl) levels. (**d**) Timeline for immunomodulatory treatments. Chloroquine: 400 mg daily, no effect. Methotrexate: 20 mg weekly, no effect. Azathioprine: 50 mg daily, stopped after concomitant viral infection was observed. Infliximab: 400 mg every 2 months, no effect. Adalimumab: 40 mg every 2 weeks, no effect. IVIG: 40 g every month, no effect. Canakinumab: 4 mg/kg every month, persistent flares but less severe in symptoms and allowed corticosteroid taper. Corticosteroids: until 2017 the patient had prescription of 60 mg for short periods (2–3 days but sometimes to 10 days) and then stopped. Since 2017, 60 mg daily of prednisone was started after a severe flare but Canakinumab allowed tapering the dose of Corticosteroids to 10 mg daily. Arrow indicates time point of serum level cytokine analysis (results shown in Fig. 2(a)). (**e**) Next-generation sequencing (NGS) results and family pedigree show presence of c.478G > A transition encoding the NLRC4 (A160T) homozygous mutation in the patient (indicated by arrow). Healthy parents and brother were heterozygous for the identified mutation. Circle represents female subjects, square represents male subjects, and solid form represents affected individual. (**f**) The structure of inactive murine NLRC4 (PDB: 4KXF) indicating residues affected by dominantly inherited mutations V341, H443, S171 (green), or A160 (orange) proximal to the nucleotide binding site (nucleotide in magenta, Leucine-rich repeat domain (LRR, grey), NACHT domain (blue, containing nucleotide-binding domain (NBD), helical domain (HD) 1 and 2, winged-helix domain (WHD)

A 60-year-old Brazilian female patient was evaluated for recurrent episodes of systemic inflammation since six months of age. Episodes were characterized by recurrent low-grade fever, chills, oral ulceration, uveitis, arthralgia, and abdominal pain followed by diarrhea with mucus and variable erythematous macular rash. Several inflammatory episodes were triggered by gastrointestinal and urinary tract infections, mainly caused by *E. coli* or both *E. coli* and *Klebsiella pathogens*, respectively. The flares lasted from 7 to 10 days and occurred on average 3–4 times a year, but more than 10 flares a year could be observed. Episodes of fever correlated with increased serum levels of C-reactive protein (CRP), elevated erythrocyte sedimentation rate (ESR), and increased serum amyloid A (SAA) (Fig. 1a). A whole-body PET-CT scan realized during one of the flares while the patient suffered from abdominal pain and diarrhea (in 2016) depicted heterogeneous and diffuse bowel glycolytic activity (Fig. 1b). Colon biopsies were performed and showed unspecific lymphocyte infiltration. However ulcers, abscesses, or clear signs of inflammation could not be observed during regular macroscopical examinations. Fecal calprotectin levels, a commonly used marker to monitor neutrophil migration into gastrointestinal tissue [7], measured 1294 mg/kg before anti-IL-1β therapy was started, which is above normal range (< 50 mg/kg) and indicates gastrointestinal inflammation. Serum IgG was decreased, while IgA and IgM levels were low but within reference ranges (Fig. 1c). Flow cytometry analysis of total lymphocytes collected when the patient was not in flare showed decreased levels of B lymphocytes (CD19 + : patient 1.6% vs. reference 11%), as well as low B memory cells (CD27 + : patient 21.6% vs. reference > 27%) and elevated double negative T cells (CD4-/CD8-/TCR αβ: patient 2.1% vs. reference < 1.7%) (data not shown). Antinuclear antibody (ANA) testing fluctuated between negative (1:80) and positive (1:320) values (data not shown). The patient had a long history of corticosteroids use on demand and did not achieve control of clinical symptoms using any disease-modifying antirheumatic drugs (DMARDS), anti-TNF therapies or intravenous immunoglobulin (IVIG). Since 2017, due to the frequency and severity of flares, high doses of corticosteroids were prescribed and somewhat effective in controlling disease (Fig. 1d). Perhaps associated with prolonged use of corticosteroids the patient developed diabetes, hypertension and dyslipidaemia. High levels of SAA were observed between the flares. As the patient had a decrease in renal function, a kidney biopsy was performed and negative congo red staining ruled out renal amyloidosis (data not shown). Because Anakinra is not available in Brazil, anti-IL-1β therapy with Canakinumab was started and able to partially control the symptoms, which resulted in reduced number of flares, diminished hospitalization, allowance for corticosteroid dose reduction, and an overall improved quality of life.

**Fig. 2:**
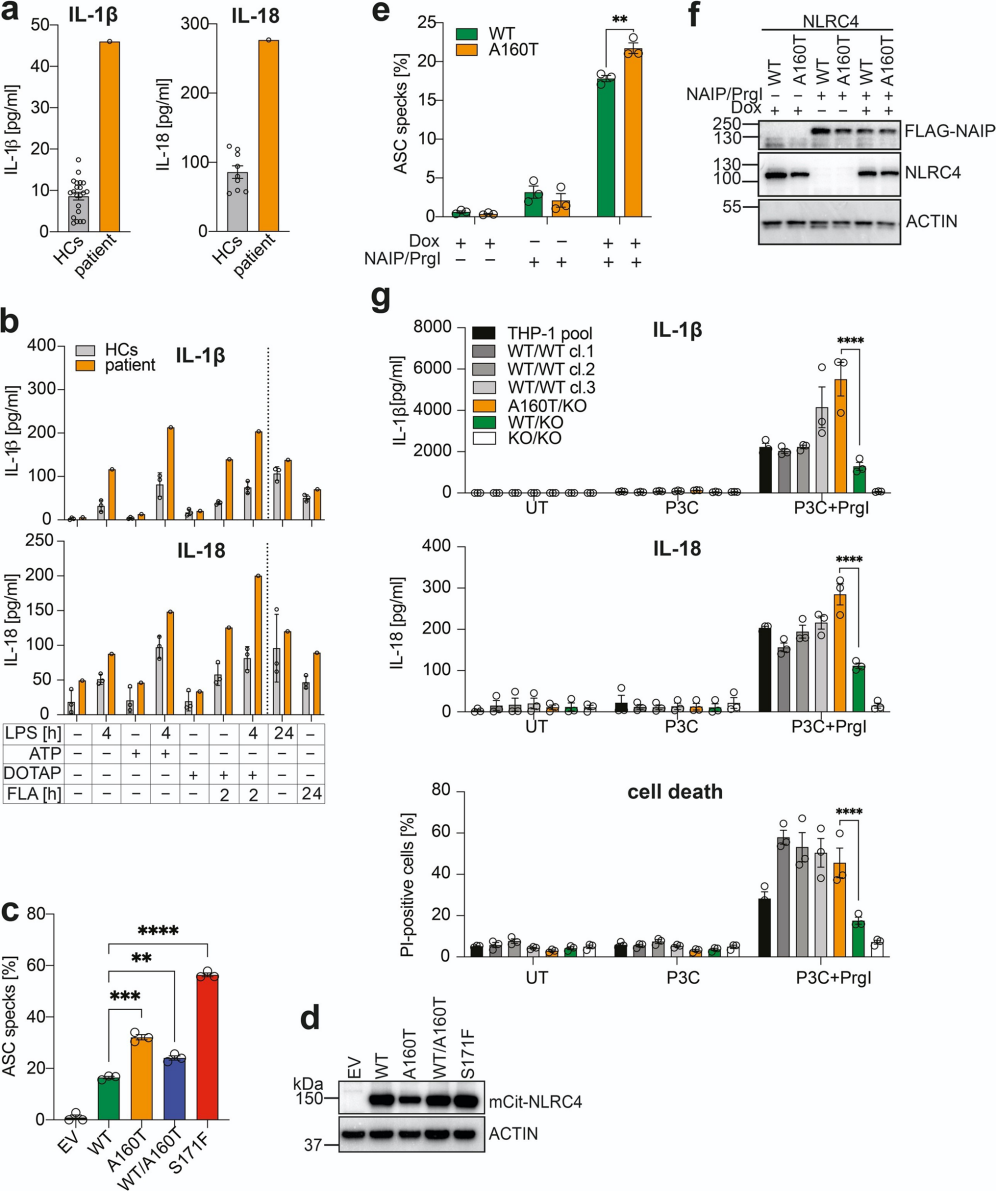
Serum cytokine levels and in vitro analysis of NLRC4 (A160T) inflammasome activation. (**a**) Analysis of patient serum cytokine levels while on anti-IL-1β treatment reveals elevated levels of IL-1β and IL-18 (healthy controls, HCs *n* = 21 for IL-1β, *n* = 9 for IL-18). (**b**) Monocyte-derived macrophages from patient and HCs (*n* = 3) were challenged with LPS (1 µg/ml) or *S. typhimurium* flagellin (FLA, 5 µg/ml) for indicated time periods and stimulated with ATP (1 mM, 15 min) or transfected FLA in DOTAP liposomes (2 h). Supernatants were analyzed for IL-1β and IL-18 by ELISA. Error bars represent SD. (**c**) Time of flight inflammasome analysis of HEK 293T-ASC-RFP cells transiently transfected with mCitrine-tagged WT, A160T, WT/A160T (heterozygous), and S171F hNLRC4 (50 ng DNA/500 μl). ASC speck formation was quantified by flow cytometry after 15 h incubation. Data are pooled from 3 independent experiments. Error bars represent SEM, statistics assessed by Student’s *t*-test. (**d**) Western blot analysis of HEK293T-ASC-RFP cells in (**c**) shown as 1 representative result of 3 independent experiments. (**e**) Flp-In 293 T-REx ASC-EGFP NLRC4 cell lines were treated with doxycycline (Dox) (1 μg/ml) to induce hNLRC4 WT or A160T expression and transiently transfected with FLAG-hNAIP and myc-PrgI (250 ng DNA/500 μl). ASC speck formation was assessed by flow cytometry 16 h post transfection. Data are shown as mean ± SEM and were pooled from *n* = 3 independent experiments. Statistical significance was assessed by two-way ANOVA with Šidák’s multiple comparison test. (**f**) Representative western blot of (**e**), *n* = 1. (**g**) Monoclonal THP-1 cell lines of indicated genotypes were analyzed for IL-1β and IL-18 release as well as cell death after 24 h treatment with Pam3CSK4 (P3C, 100 ng/ml) and PrgI (1 ml retroviral supernatant) by ELISA or flow cytometry, respectively. Data were pooled from 3 independent experiments and shown as mean ± SEM. Statistical testing by two-way ANOVA with Tukey’s multiple comparison test was performed. Only statistical significances relevant for the study results are indicated. *P < 0.05, **P < 0.01, ***P < 0.001, ****P < 0.0001

Analysis of a target gene panel [3] and subsequent next generation sequencing (NGS) identified a homozygous mutation in exon 4 of the *NLRC4* gene (NM_021209), c.478G > A encoding the p.A160T variant. Familial genetic segregation analysis using standard Sanger sequencing identified the healthy father, mother, and brother to be heterozygous carriers of the mutant allele (Fig. 1e). Parents were non-consanguineous, with the father of German ancestry and the mother of Syrian ancestry. Heterozygous occurrence of this allele in the general population has been reported with an estimated allele frequency 0.00081, but there are no reported healthy homozygotes in the Genome Aggregation Database (GnomAD).

To investigate potential structural consequence of the p.A160T substitution, we analyzed this residue in the crystal structure of inactive murine NLRC4 (PDB:4KXF), since no structure of human NLRC4 is available to date and proteins from both species share 75% identity on amino acid sequence level (NCBI blastp alignment). A160 is a conserved residue between both species and in the inactive structure of mNLRC4 accessible on the surface and located proximal to the nucleotide binding site and residues p.V341, p.H443, p.S171, where pathogenic dominant mutations have been identified [2, 4, 8, 9] (Fig. 1f). Whereas NLRC4-AID-associated dominantly inherited mutations are buried in the structure with direct links to the nucleotide binding site, the novel recessive mutation is solvent exposed. This could reflect its milder phenotype and the necessity to have two alleles causing a Mendelian disease. Although it is not currently known how the dominant mutations at this site promote an open, active conformation of NLRC4, the fact that A160T is adjacently located is a useful observation.

In contrast to other inflammasomopathies that are mainly driven by increased release of IL-1β, IL-18 was suggested to be the main cytokine responsible for the clinical manifestations of NLRC4-AID [10]. Patient serum cytokine levels during anti-IL-1β treatment with Canakinumab evidenced increased levels of IL-18 (276.80 pg/ml) compared to healthy controls (HCs) (mean 85.82 pg/ml) and a remaining slight increase in IL-1β levels (patient 46 pg/ml, healthy controls mean 8.62 pg/ml) (Fig. 2a). At the time of serum collection for cytokine measurements, the patient had already received continuous Canakinumab treatment for 8 months (1 injection of 4 mg/kg per month). Serum was collected just before another dose of Canakinumab was injected. Since no patient data on cytokine serum levels prior to Canakinumab treatment are available and Canakinumab binding to circulating IL-1β has been reported to prolong cytokine half-life [11], the interpretation of serum IL-1β levels remains indicative. Nonetheless, elevated serum IL-18 levels that are independent of Canakinumab treatment suggest increased NLRC4 inflammasome activation. In order to investigate the disease-causing potential of the mutation, patient monocyte-derived macrophages (MDMs) were stimulated with lipopolysaccharide (LPS), a Toll-like receptor (TLR) 4 agonist, and then ATP or transfected flagellin to activate NLRP3 and NLRC4 inflammasome formation, respectively (Fig. 2b). Treatment with LPS and cytoplasmic flagellin alone did reveal some differences in IL-1β and IL-18 release, suggesting increased baseline and ligand-induced activity of NLRC4 A160T in patient MDMs without LPS-priming (Fig. 2b). However, substantially elevated IL-1β and IL-18 levels were observed in patient-derived cells upon activation of both investigated inflammasomes, with the most pronounced difference in IL-18 released following specific stimulation of NLRC4 in primed cells (LPS + DOTAP/FLA) (Fig. 2b). TNF levels, measured as inflammasome-independent priming control, were comparable in all tested conditions (Suppl. Figure 1a). Interestingly, we found that longer-term 24 h stimulation with LPS did not alter IL-18 production, but 24 h FLA (extracellular) showed an increase in IL-18 release for the patient sample, which may suggest internalization of FLA and an increased response of NLRC4 A160T at later time points. Overall, these results suggest NLRC4 A160T autoactivation to some extent, and that environment, infection, or other triggers are able to exaggerate responses due to NLRC4 A160T, which is consistent with flares of disease experienced by the patient. Increased responsiveness of MDMs from another NLRC4-MAS patient to NLRP3 stimulation (LPS + ATP) has been reported in the literature [1] and studies have suggested an interaction of NLRP3 with NLRC4 and recruitment to the same inflammasome complex [12, 13], which could provide a mechanistic basis to explain why NLRP3 stimuli also appear to trigger increased responses in NLRC4 A160T MDMs; however, this explanation remains speculative.

As an in vitro readout for inflammasome assembly and activation on a single cell level, formation of apoptosis-associated speck-like protein containing a CARD (ASC) specks was quantified by flow cytometry [5]. HEK293T cells stably expressing ASC-RFP were transiently transfected with mCitrine-tagged NLRC4 wild type (WT) or A160T. Increased ASC speck formation was observed with NLRC4 A160T suggesting spontaneous inflammasome assembly. To resemble a heterozygous carrier, WT and A160T NLRC4 were co-transfected at a 50:50 ratio, which resulted in slightly elevated ASC speck formation compared to WT NLRC4. The dominant mutation NLRC4 S171F [4, 8] results in stronger inflammasome activation than A160T, which is recessively inherited in this case (Fig. 2c). Protein levels of NLRC4 WT, WT/A160T, and S171F are comparable following overexpression in this assay, but slightly reduced for NLRC4 A160T (Fig. 2d). This finding was consistent between experimental repeats, which may suggest that in some circumstances, this mutant is less stable and degraded more rapidly.

To independently verify these results, Flp-In 293 T-REx cell lines stably expressing ASC-EGFP and NLRC4 WT, A160T, or S171F were generated (Suppl. Figure 1b). Site-specific genomic integration of a single copy of NLRC4 under a tetracycline-inducible promoter yielded a highly controlled and consistent experimental system. Importantly, western blot analysis confirmed similar expression levels between different cell lines (Suppl. Figure 1c), which leads us to speculate that reduced A160T stability might only occur when expressed above endogenous levels (Fig. 2d). Without stimulation, ASC specking was induced by autoactivating mutation S171F, but not by A160T or WT NLRC4 (Suppl. Figure 1d). Instead, for this system, increased ASC-speck formation by NLRC4 A160T over WT was only observed following NLRC4 activation by transient transfection of the sensor protein NAIP and *S.typhimurium* type three secretion system (T3SS) needle protein PrgI-expressing plasmids (Fig. 2e, f). However in this experiment, protein expression levels of NLRC4 A160T were slightly below WT levels (Fig. 2f). This was attributable to a small difference in mRNA transcript expression in this case (qRT-PCR data not shown), rather than intrinsic defects in protein stability which may result is slight underestimation of the effect caused by NLRC4 A160T.

To recapitulate the effect of NLRC4 A160T in a human monocyte-like cell line with endogenous expression of proteins relevant for inflammasome activation and cytokine release, we employed CRISPR/Cas9-mediated gene editing and homology-directed repair to genetically modify THP-1 cells and introduce the A160T mutation on genomic level (Suppl. Figure 2a). Monoclonal cell lines were generated and sequences of the *NLRC4* genomic locus as well as mRNA transcripts confirmed by Sanger sequencing (Suppl. Figure 2b). This experimental approach yielded one clone with in-frame insertion of the correct mutation (c.478G > A) on one allele and 1 bp deletion on the second allele, generating an A160T/KO genotype. A matching WT/KO control clone and 3 WT/WT clones carrying one or two functional *NLRC4* WT alleles, respectively, were selected as controls for subsequent stimulation experiments. Furthermore, a KO/KO clone with homozygous 143 bp deletion inducing a premature stop codon was included (Suppl. Figure 2b). qRT-PCR analysis of *NLRC4* transcript levels demonstrates that expression in A160T/KO and WT/KO clones is around 50% reduced compared to THP-1 pool and WT/WT clones with 2 functional NLRC4 alleles (Suppl. Figure 2c). Therefore, the WT/KO clone was used as comparator group in subsequent stimulation assays.

NLRC4 stimulation of monoclonal cell lines with PrgI and TLR1/2 agonist Pam3CSK4 (P3C)-mediated priming suggests enhanced inflammasome signalling by NLRC4 A160T (clone A160T/KO) as observed by significantly elevated release of IL-1β and IL-18 into supernatants and increased cell death. Spontaneous signalling after P3C-priming alone was not observed, indicating that A160T does not cause NLRC4 autoactivation and requires a NLRC4-specific stimulus to elicit pathogenicity (Fig. 2g).

To exclude clonal differences between these THP-1 cell lines, we tested inflammasome priming by quantification of IL-8, a NF-κB-induced cytokine (Suppl. Figure 2d), and activation of a different inflammasome sensor, NLRP3 (Suppl. Figure 2e). Small differences were observed in IL-8 levels in response to P3C priming alone, however that was not consistent in the PrgI-treated samples (Suppl. Figure 2d). Cells stimulated with P3C and Nigericin, to activate NLRP3, also had smaller differences in IL-1β and IL-18 production but induced cell death did not differ (Suppl. Figure 2e). This data suggests that there are likely some clonal differences between the cell lines, but not to the same extent as for NLRC4 activation.

Unlike IL-1β, which is a NF-κB-induced cytokine, the IL-18 precursor protein is constitutively expressed in various cell types, including intestinal epithelial cells and was previously shown to be involved in intestinal inflammation [14]. Since the severity of inflammatory bowel disease (IBD) was correlated with increased IL-18 secretion in clinical studies [14], we aimed to investigate the genome-wide association of NLRC4 (A160T) with Crohn’s disease (CD) and ulcerative colitis (UC), the two common forms of IBD. Using data from the IBD exomes portal (Broad institute), we found significant enrichment of the NLRC4 (A160T) allele in UC patients compared to healthy controls, with an odds ratio (OR) of 2.546 ((95% 1.778–3.644), *P* = 0.01305) (Table 1). Other genetic variants have been reported to be associated with UC, with odds ratios comparable to NLRC4 (A160T). These include variants in inflammatory pathway related molecules, such as NLRP7 (S361L): OR = 4.79, *P* = 0.0039 [15] and NOD2 (R38M): OR = 2.904, *P* = 0.03291 (IBD exomes portal). Of note, the correlation between NLRC4 (A160T) and CD was less clear (OR = 1.62 (95% 1.119–2.346), *P* = 0.2615), suggesting this locus as a risk factor predominantly for the development of UC.

**Table 1:** Association of *NLRC4* (c.478G > A, p.A160T) with ulcerative colitis (UC) in the genome across different populations. Data accessed from IBD Exomes Portal, Cambridge, MA http://ibd.broadinstitute.org, accessed October 2018

Population	Homozygous reference	Heterozygous alternate	UC allele frequency	Non-UC allele frequency	OR [95% interval]	*P-*value
European (Ashkenazi-Jewish)	5680	5	0.0007184	0.0003292	1.035	0.9749
Non-Finnish European	7726	14	0.001161	0.000361	2.862	0.1707
European (Finnish)	11,453	26	0.003589	0.001159	4.125	0.0392
**Total**					**2.546** **[1.778, 3.644]**	**0.01305**

In summary, *NLRC4* (c.478G > A, p.A160T) is likely to be the first recessive mutation in this gene, causing a monogenic disease characterized by autoinflammation and immune dysregulation following activation by environmental triggers. This allele is pathogenic in vitro and enhances stimulus-dependent NLRC4 signalling, however with reduced strength compared to dominant mutations in NLRC4, and results in a modest increase in IL-18 in vivo. Nevertheless, carriers with NLRC4 (A160T) are at increased risk of developing ulcerative colitis.

## Supplementary Information

Below is the link to the electronic supplementary material.

Supplementary file1 (DOCX 785 KB)

## Data Availability

Source data and materials are available upon request from the authors.

## References

[1] Canna SW, de Jesus AA, Gouni S, Brooks SR, Marrero B, Liu Y (2014). An activating NLRC4 inflammasome mutation causes autoinflammation with recurrent macrophage activation syndrome. Nat Genet..

[2] Romberg N, Al Moussawi K, Nelson-Williams C, Stiegler AL, Loring E, Choi M (2014). Mutation of NLRC4 causes a syndrome of enterocolitis and autoinflammation. Nat Genet..

[3] Papa R, Rusmini M, Volpi S, Caorsi R, Picco P, Grossi A (2020). Next generation sequencing panel in undifferentiated autoinflammatory diseases identifies patients with colchicine-responder recurrent fevers. Rheumatology (Oxford)..

[4] Moghaddas F, Zeng P, Zhang Y, Schutzle H, Brenner S, Hofmann SR (2018). Autoinflammatory mutation in NLRC4 reveals a leucine-rich repeat (LRR)-LRR oligomerization interface. J Allergy Clin Immunol..

[5] Sester DP, Thygesen SJ, Sagulenko V, Vajjhala PR, Cridland JA, Vitak N (2015). A novel flow cytometric method to assess inflammasome formation. J Immunol..

[6] Miao EA, Mao DP, Yudkovsky N, Bonneau R, Lorang CG, Warren SE (2010). Innate immune detection of the type III secretion apparatus through the NLRC4 inflammasome. Proc Natl Acad Sci U S A..

[7] Pathirana WGW, Chubb SP, Gillett MJ, Vasikaran SD (2018). Faecal Calprotectin. Clin Biochem Rev..

[8] Liang J, Alfano DN, Squires JE, Riley MM, Parks WT, Kofler J (2017). Novel NLRC4 Mutation causes a syndrome of perinatal autoinflammation with hemophagocytic lymphohistiocytosis, hepatosplenomegaly, fetal thrombotic vasculopathy, and congenital anemia and ascites. Pediatr Dev Pathol..

[9] Kitamura A, Sasaki Y, Abe T, Kano H, Yasutomo K (2014). An inherited mutation in NLRC4 causes autoinflammation in human and mice. J Exp Med..

[10] Weiss ES, Girard-Guyonvarc'h C, Holzinger D, de Jesus AA, Tariq Z, Picarsic J (2018). Interleukin-18 diagnostically distinguishes and pathogenically promotes human and murine macrophage activation syndrome. Blood..

[11] Lachmann HJ, Lowe P, Felix SD, Rordorf C, Leslie K, Madhoo S (2009). In vivo regulation of interleukin 1beta in patients with cryopyrin-associated periodic syndromes. J Exp Med..

[12] Qu Y, Misaghi S, Newton K, Maltzman A, Izrael-Tomasevic A, Arnott D (2016). NLRP3 recruitment by NLRC4 during Salmonella infection. J Exp Med..

[13] Man SM, Hopkins LJ, Nugent E, Cox S, Glück IM, Tourlomousis P (2014). Inflammasome activation causes dual recruitment of NLRC4 and NLRP3 to the same macromolecular complex. Proc Natl Acad Sci..

[14] Pizarro TT, Michie MH, Bentz M, Woraratanadharm J, Smith MF, Foley E (1999). IL-18, a novel immunoregulatory cytokine, is up-regulated in Crohn's disease: expression and localization in intestinal mucosal cells. J Immunol..

[15] Onoufriadis A, Stone K, Katsiamides A, Amar A, Omar Y, de Lange KM (2018). Exome sequencing and genotyping identify a rare variant in NLRP7 gene associated with ulcerative colitis. J Crohns Colitis..

