# Supplementary material for: Recessive NLRC4-Autoinflammatory Disease Reveals an Ulcerative Colitis Locus
Source: J Clin Immunol. 2021 Nov 16;42(2):325–35. doi: 10.1007/s10875-021-01175-4 (PMC8821057; doi:10.1007/s10875-021-01175-4)
Supplement: Supplementary file 1 — Supplementary file1 (DOCX 785 KB) [file 10875_2021_1175_MOESM1_ESM.docx]

**Suppl. Table 1:** **Primer, guide RNA and oligo repair template sequences used in this study.** Within the oligo repair template modified nucleotides to introduce *Bsa*I restriction enzyme recognition site (c.468C>T underlined and bold) and c.478G>A mutation encoding p.A160T (italic and bold) are highlighted.

|  | **Sequence (5’🡪3’)** |
| --- | --- |
| CRISPR/Cas9 genome editing | |
| *NLRC4*-specific guide RNA | GACCCTGAATGGCCTCCTGC |
| oligo repair template | GACCAACACCATCACCGCGTGGAGCAGCTGACCCTGAATGG**T**CTCCTGCAG***A***CTCTTCAGAGCCCCTGCATCATTGAAGGGGAATCTGGC |
| Mutagenesis primer |  |
| *hNLRC4*_A160T_mut_F | CTCCTGCAGACTCTTCAGAGCC |
| *hNLRC4*_A160T_mut_R | GGGCTCTGAAGAGTCTGCAGGAG |
| *hNLRC4*_S171F_mut_F | TTGCCTTTGCCAAATTCCCCTTCAATGATGCAGGGG (4) |
| *hNLRC4*_S171F_mut_R | CCCCTGCATCATTGAAGGGGAATTTGGCAAAGGCAA (4) |
| Amplification of genomic *NLRC4* locus | |
| *hNLRC4*_F_genomic DNA | GTAAAGGATGCTGGGGAAAG |
| *hNLRC4*_R_genomic DNA | GCTTGAATTCATTGTAGCCATC |
| Amplification of *NLRC4* mRNA transcript for Sanger sequencing | |
| *hNLRC4*_F_cDNA | GGACAAAGTCTTTTTCATCAGACATC |
| *hNLRC4*_R_cDNA | CAGGCCTTCAGCTAGTTTTATA |
| Sequencing |  |
| *hNLRC4*_seq | CACCAATGGCAGATTCTCAG |
| qRT-PCR | |
| *hNLRC4*_qRT-PCR_F | CATCATTTGCTGCGAGAAGGTGG |
| *hNLRC4*_qRT-PCR_R | CGTCCAAGTCTCCTTCTGATGTCTG |
| *hACTB*_qRT-PCR_F | GCGAGAAGATGACCCAGATC |
| *hACTB*_qRT-PCR_R | CCAGTGGTACGGCCAGAGG |


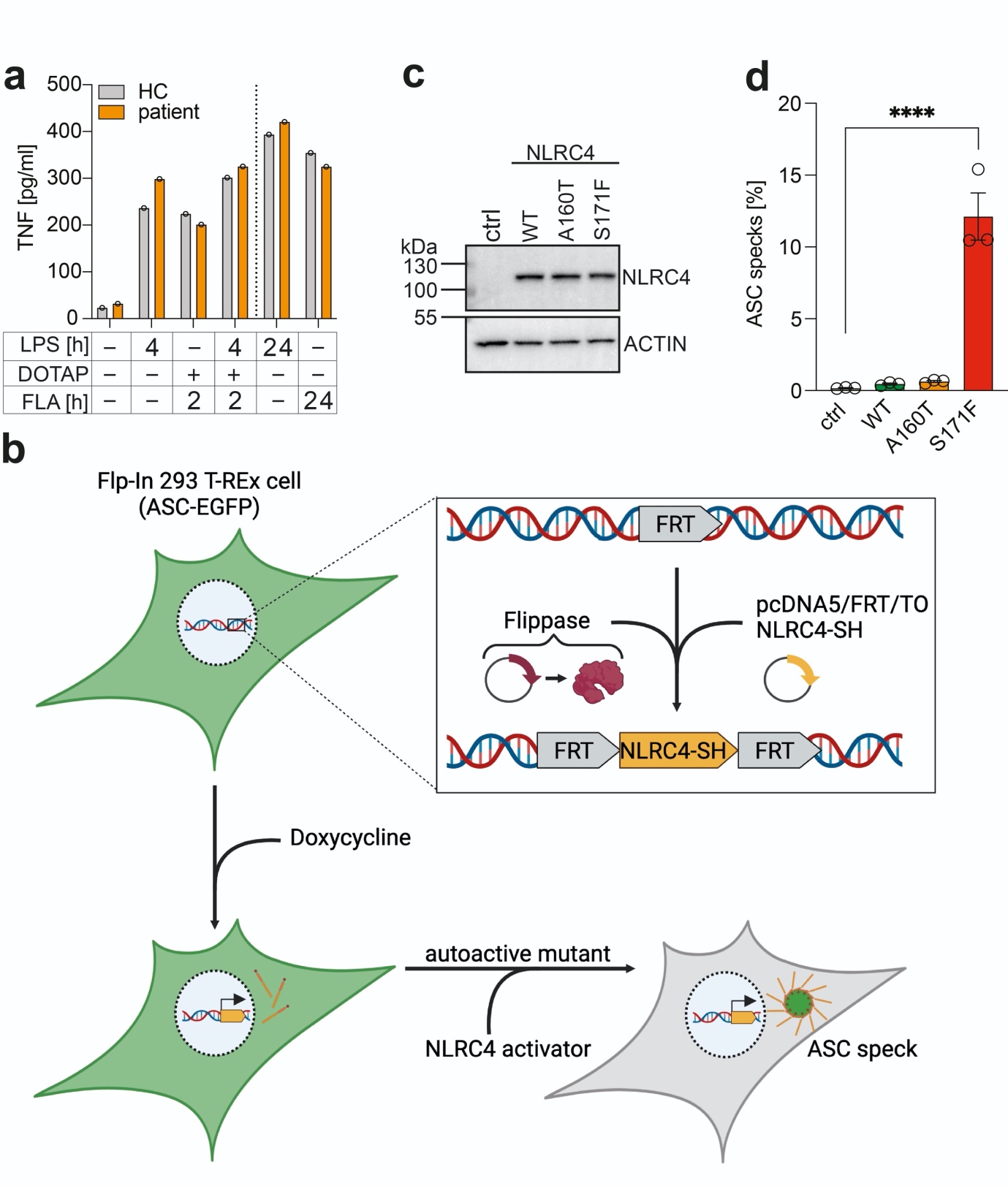


**Supplementary Figure 1: MDM-released TNF levels and inflammasome formation in Flp-In 293 T-REx cell lines with stable isogenic NLRC4 expression.** (**a**) ELISA-quantification of TNF levels in monocyte-derived macrophages (MDM) from the patient and one healthy donor (HC) stimulated with LPS (1 µg/ml) or *S.typhimurium* flagellin (FLA, 5 µg/ml) for the indicated time (hours (h)). Cytoplasmic FLA was delivered in DOTAP liposomes for 2 h. (**b**) Generation of NLRC4-expressing Flp-In 293 T-REx ASC-EGFP cell lines. Transient co-transfection of Flp-In 293 T-REx cells stably expressing ASC-EGFP with the Flippase recombinase-expressing plasmid (pOG44) and an expression plasmid (pcDNA5/FRT/TO NLRC4-SH) encoding the Strep2-HA (SH)-tagged NLRC4 gene flanked by Flippase recognition sites (FRT) generates stable isogenic cell lines. Doxycycline treatment induces NLRC4-SH transcription and subsequent ASC specking of NLRC4 carrying an autoactivating mutation or following stimulation with NLRC4-specific activators. Figure has been created with Biorender.com. (**c**) Representative western blot of experiment shown in (d). NLRC4 expression levels in Flp-In 293 T-REx ASC-EGFP NLRC4-SH expressing cells after doxycycline treatment (1 μg/ml) for 27 h. Representative shown, n=3. (**d**) ASC speck formation analysed by flow cytometry following doxycycline treatment (1 μg/ml) for 27 h of NLRC4 WT, A160T or S171F Flp-In 293 T-REx ASC-EGFP cell lines. Control (ctrl) cells did not contain NLRC4 insert. Data pooled from 3 independent experiments shown as mean ± SEM. Statistical significance was assessed by one-way ANOVA*.* *****P*<0.0001.


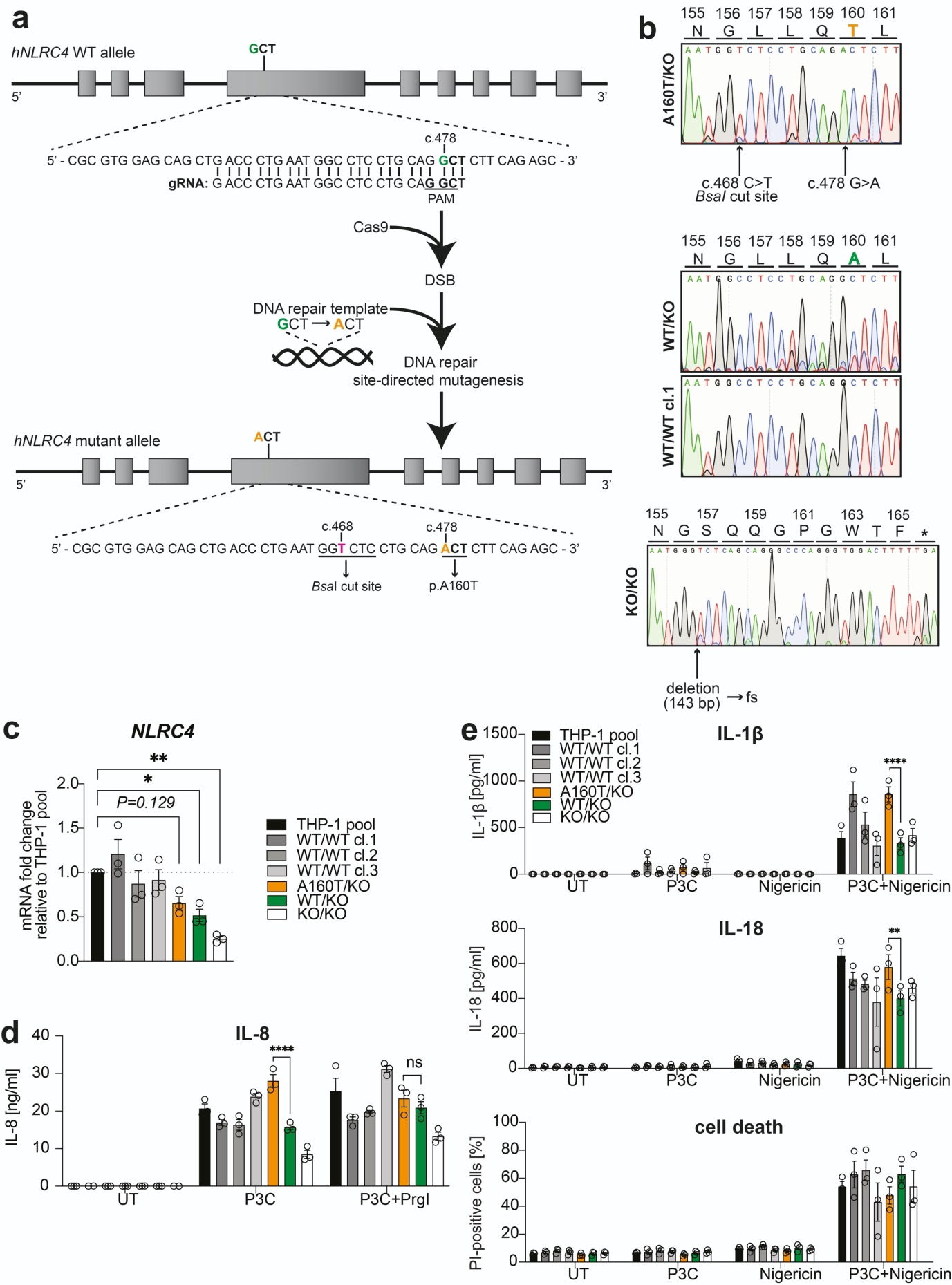


**Supplementary Figure 2: Generation and validation of monoclonal THP-1 cell lines. (a)** CRISPR/Cas9-mediated homology-directed repair was employed to introduce the A160T mutation (encoded by c.478G>A) on genomic level. CRISPR guide binding site within exon 4, reverse PAM site (underlined, bold) and sequence of the used guide RNA (gRNA) are shown. The WT sequence c.478G is highlighted in green (bold) and was exchanged to c.478A (orange) encoded in the DNA repair template. Additionally, a silent mutation at c.468C>T was introduced to generate a *Bsa*I restriction enzyme recognition site, which was subsequently used to screen monoclonal cell lines for repair template integration. **(b)** Sanger sequencing following RNA isolation, cDNA synthesis and *NLRC4*-specific PCR confirmed presence of c.478G>A (p.A160T) and c.468C>T in the actively transcribed *NLRC4* mRNA transcript in THP-1 cell clone A160T/KO. WT/KO and WT/WT clone (cl.) 1. are shown as reference for the WT sequence. Amino acid position and corresponding residues are indicated. Arrows indicate relevant mutations. A homozygous 143 bp deletion was detected in the KO/KO cell line, inducing a frameshift (fs) and premature stop codon at p.166. **(c)** qRT-PCR analysis of *NLRC4* mRNA transcript levels in THP-1 cell clones with indicated genotypes shown as mean ± SEM of 3 independent experiments. *P*-values were calculated using one-way ANOVA and THP-1 pool was used as comparator group. **(d)** Quantification of released IL-8 levels (ng/ml) by ELISA in THP-1 cell lines after 24 h stimulation with Pam3CSK4 (P3C, 100 ng/ml) and PrgI retroviral supernatant. Data were pooled from 3 independent experiments and presented as mean ± SEM. Statistical analysis: two-way ANOVA with Tukey’s multiple comparison test. ns, not significant. **(e)** Assessment of IL-1β, IL-18 release and cell death following NLRP3 stimulation by ELISA or flow cytometry, respectively. THP-1 cell clones were primed with P3C (100 ng/ml) for 3 h with subsequent Nigericin treatment (10 μM, 1 h). Data were pooled from 3 independent experiments and shown as mean ± SEM. Statistical testing by two-way ANOVA. Only statistical significances relevant for the study results are indicated. **P* < 0.05, ***P* < 0.01, ****P*<0.001, *****P*<0.0001.
